# Dysregulation of the Descending Pain System in Temporomandibular Disorders Revealed by Low-Frequency Sensory Transcutaneous Electrical Nerve Stimulation: A Pupillometric Study

**DOI:** 10.1371/journal.pone.0122826

**Published:** 2015-04-23

**Authors:** Annalisa Monaco, Ruggero Cattaneo, Luca Mesin, Eleonora Ortu, Mario Giannoni, Davide Pietropaoli

**Affiliations:** 1 University of L’Aquila, Department of Life, Health and Environmental Sciences, Building Delta 6 Dental Unit, St Salvatore Hospital—Via Vetoio 67100 L’Aquila, Italy; 2 Department of Electronics and Telecommunications, Politecnico di Torino, Turin, Italy; University of Würzburg, GERMANY

## Abstract

Using computerized pupillometry, our previous research established that the autonomic nervous system (ANS) is dysregulated in patients suffering from temporomandibular disorders (TMDs), suggesting a potential role for ANS dysfunction in pain modulation and the etiology of TMD. However, pain modulation hypotheses for TMD are still lacking. The periaqueductal gray (PAG) is involved in the descending modulation of defensive behavior and pain through μ, κ, and δ opioid receptors. Transcutaneous electrical nerve stimulation (TENS) has been extensively used for pain relief, as low-frequency stimulation can activate µ receptors. Our aim was to use pupillometry to evaluate the effect of low-frequency TENS stimulation of μ receptors on opioid descending pathways in TMD patients. In accordance with the Research Diagnostic Criteria for TMD, 18 females with myogenous TMD and 18 matched-controls were enrolled. All subjects underwent subsequent pupillometric evaluations under dark and light conditions before, soon after (end of stimulation) and long after (recovery period) sensorial TENS. The overall statistics derived from the darkness condition revealed no significant differences in pupil size between cases and controls; indeed, TENS stimulation significantly reduced pupil size in both groups. Controls, but not TMD patients, displayed significant differences in pupil size before compared with after TENS. Under light conditions, TMD patients presented a smaller pupil size compared with controls; the pupil size was reduced only in the controls. Pupil size differences were found before and during TENS and before and after TENS in the controls only. Pupillometry revealed that stimulating the descending opioid pathway with low-frequency sensory TENS of the fifth and seventh pairs of cranial nerves affects the peripheral target. The TMD patients exhibited a different pattern of response to TENS stimulation compared with the controls, suggesting that impaired modulation of the descending pain system may be involved in TMD.

## Introduction

Recent literature has suggested that TMD patients may suffer from dysfunction in the brain network that supports sensory, pain, emotional, and cognitive processes [1–8]. Some authors have focused on the dysregulation of the autonomic nervous system (ANS) in TMD patients [9–13], suggesting that TMD could be the clinical manifestation of multisystem dysregulation [14].

Among the structures that are involved in the brain network regulating the sensory, pain, emotional, and cognitive systems, the periaqueductal gray (PAG) has a key role. It receives afferent connections from cortical areas associated with cognition and motivation related to sensory and pain perception [15–17], and it then projects the connections to the centers controlling the peripheral afferent inputs and couples autonomic reactions in a specific manner [18,19].

The role of the PAG in descending pain control via endogenous opioids is one of the most studied pathways [20–22], and several findings suggest that chronic pain is promoted by an abnormal modulation of the descending endogenous pain system [23,24].

Transcutaneous electric nerve stimulation (TENS) has been used for a long time to relieve pain [25–27]. Its main effect is believed to be achieved through the modulation of descending inputs from the ventral-lateral PAG to the rostroventral medial medulla (RVM) [28,29]. In particular, low-frequency TENS is suggested to activate μ opioid receptors [30–32].

Bilateral low-frequency TENS of the fifth and seventh cranial nerves has been suggested to treat TMD [33–36] on the basis of the resulting pain relief [37,38] monitored by surface electromyography (EMG) of masticatory muscles and kinesiography of jaw movements [39,40]. However, despite the clinical relevance of TENS in TMD, the above-cited studies focused on clinical pain or jaw muscle effects. Therefore, no information can be drawn regarding the hypothesis of autonomous or central dysfunction in patients with TMD.

Recent findings promote the use of pupillometry to study the effects of opioids in opioid-maintained patients and healthy subjects [41–43]. Moreover, the dysregulation of the ANS has been reported in TMD [44], obstructive sleep apnea syndrome [45–48], and other autonomous disorders [49–52] by pupillometry. For these reasons, pupillometry can be considered a non-invasive, sensitive and cost-effective tool for investigating, via pupil constriction (miosis), the ANS and opioid-related miosis [53–55].

According to the above-mentioned literature, it is possible that the effects of low-frequency TENS that are mediated by the supraspinal center and the endogenous opioid path can be demonstrated at the pupil level (miosis). The aim of this study was to evaluate pupillometric indexes using low-frequency TENS to test the hypothesis that the opioid descending pathway is dysregulated in patients with TMD.

## Materials and Methods

### Subjects

This study was conducted in accordance with the Declaration of Helsinki. The Committee on Ethics in Science of the University of L’Aquila, L’Aquila, Italy, approved the study, and written informed consent was obtained from each subject and electronically stored as suggested by our institutional guidelines.

### Inclusion/Exclusion Criteria

Eighteen Caucasian patients (mean age 26.5±5.3 years) who fulfilled the following criteria were included in the study group: female gender; age less than 40 years; myogenous TMD; pain duration longer than 3 months; presence of complete permanent dentition, with the possible exception of the third molars; normal occlusion. Patients were excluded from the study if they met one or more of the following criteria: presence of systemic or metabolic diseases; eye diseases or visual defects; history of local or general trauma; neurological or psychiatric disorders; muscular diseases; cervical pain; bruxism, as diagnosed by the presence of parafunctional facets and/or anamnesis of parafunctional tooth clenching and/or grinding; pregnancy; assumed use of anti-inflammatory, analgesic, anti-depressant, opioid, or myorelaxant drugs; smoking; fixed or removable prostheses; fixed restorations that affected the occlusal surfaces; and either previous or concurrent orthodontic or orthognathic treatment.

For comparison with previous literature [7,8,56,57], the diagnosis of myofascial-type TMD was provided after clinical examination by a trained clinician according to group 1a and 1b of the Research Diagnostic Criteria for TMD (RDC/TMD) [58], in a blinded manner (RC).

The control group consisted of 18 age- and gender-matched Caucasian subjects scheduled for a routine checkup at the University Clinic, without signs or symptoms of TMD, who fulfilled the inclusion and exclusion criteria (mean age 25.1±5.7 years).

**Fig 1:**
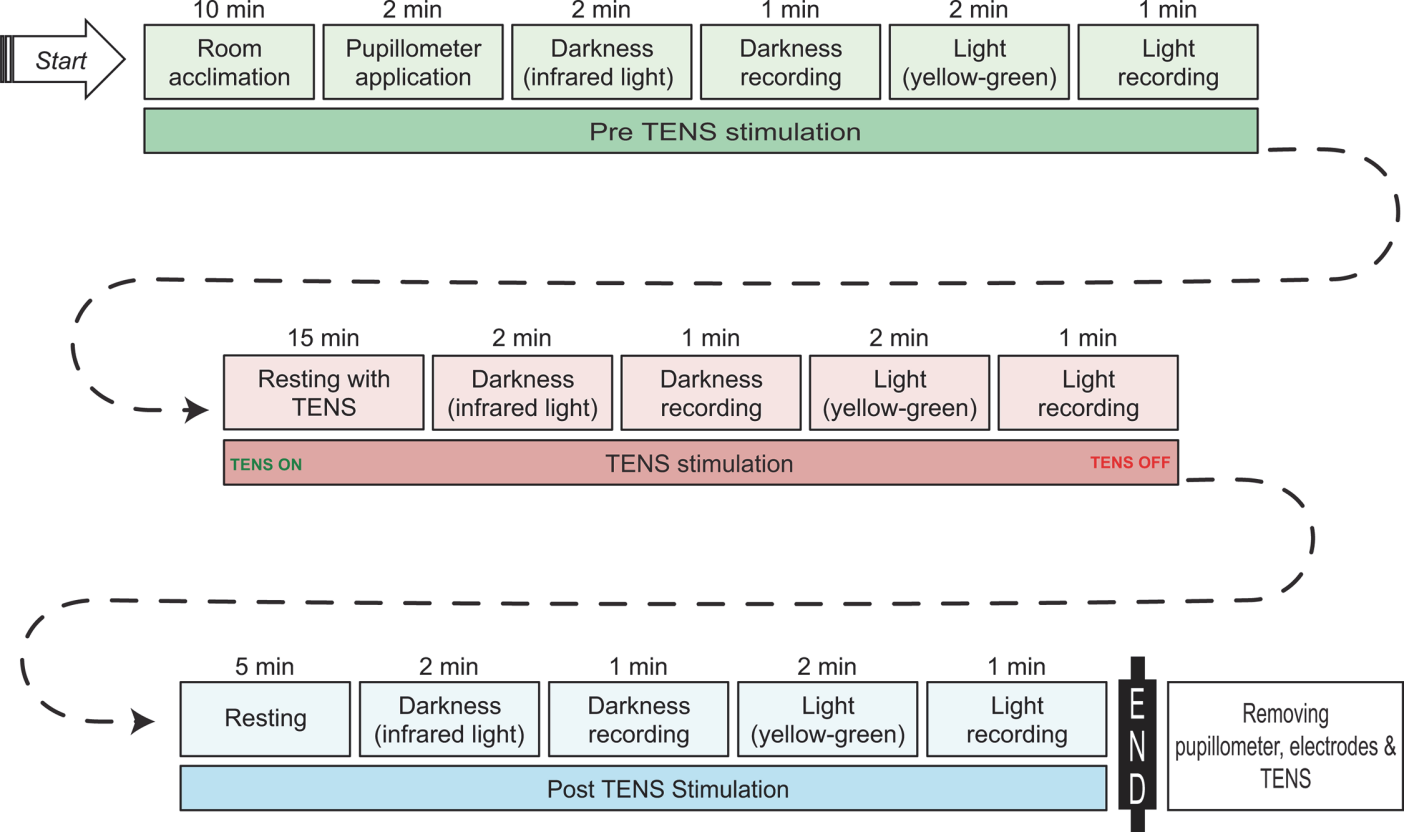
Diagram of the Experimental Protocol. Before beginning the experimental session, each enrolled subject was prepared with TENS electrodes and connected to a turned-off TENS device. Subsequently, they underwent three experimental phases: Pre TENS stimulation (green); During TENS stimulation (red); and Post TENS stimulation (blue). Pupillometer application and its removal occurred after the acclimation period and at the end of the experimental session, respectively (green). Based on the literature, central nervous system stimulation was obtained by sensory stimulation of cranial nerves V and VII with low-frequency TENS for 21 minutes (red) [28–32]. To avoid operator interference, the pupillometer, electrodes and TENS device were removed only at the end of the experimental session.

Each enrolled subject underwent the experimental protocol described in Fig 1.

### Pupillometry

Pupillometry was performed with an infrared pupillometer (Oculus system, Inventis srl, Padova, Italy), which was composed of two infrared CCD cameras (resolution of 720x576 pixels, 256 gray levels) mounted on a light helmet (1.5 kg), with a sampling frequency of 25 frames/s. To stabilize accommodation, the subjects were asked to focus their eyes on the light point in the pupillometer [42].

The assessment of pupil size was performed under light conditions, with the eyes illuminated using a yellow-green LED with a 740-nanometer wavelength, as well as under dark conditions that were obtained using only three infrared diodes with a wavelength of 880 nanometers. Pupillometric recordings were acquired in digital form and processed using the Tarjan algorithm designed to evaluate strongly connected components [59] to obtain frame-by-frame measurements of the pupil area, expressed as the number of pixels covering it. A template was positioned on the computer screen to allow for the correction of the eye position to avoid errors in the alignment of the pupils, as previously described [44].

Pupillometry was performed with the subjects in a horizontal supine position on a bed. Room temperature (21°C) and relative humidity (50%) remained constant. Any external or internal noise sources were excluded.

Before the pupillometric recording sessions, patients were invited to lie on the bed for clinical examination with their eyes open for at least 10 minutes to adapt to the temperature and humidity of the room, as well to reduce their anxiety (Fig 1). Then, the pupillometer was applied and maintained until the end of the recording session.

### Recording Procedure

All recording procedures are described in the protocol diagram (Fig 1). Briefly, after 2 minutes of darkness under an infrared light condition, 1 minute of recording was obtained, followed by the application of yellow-green light for 2 minutes and subsequent recording for 1 minute; this procedure was applied before, during and after sensory TENS stimulation, for a total of 6 pupillometric recordings of 60 seconds each (Fig 1). The recording sessions were performed by an expert operator (RC) in a blinded manner.

Based on previous literature [44], darkness and light adaptation was minimized, due to the short duration of infrared and yellow-green stimulation, and any potential, residual adaptive effect, if present, could be observed in both groups.

### Stimulation Procedure

The method for sensory TENS was described previously [60]. Briefly, a J5 Myomonitor TENS Unit device (Myotronics-Noromed, Inc., Tukwila, WA, USA) with disposable electrodes (Myotrode SG Electrodes, Myotronics-Noromed, Inc., Tukwila, WA, USA) was used. This low-frequency neurostimulator generates a repetitive synchronous and bilateral stimulus delivered at 1.5-s intervals, with an adjustable amplitude of approximately 0–24 mA, a duration of 500 μs, and a frequency of 0.66 Hz. The two TENS electrodes were placed bilaterally over the cutaneous projection of the notch of the fifth pair of cranial nerves, which was located between the coronoid and condylar processes and was retrieved by manual palpation of the zone anterior to the tragus; a third grounding electrode was placed in the center of the back of the neck.

Based on the literature, central nervous system stimulation was obtained by sensory stimulation of cranial nerves V and VII with low-frequency TENS for 21 minutes [28–32]. The amplitude of TENS stimulation started at 0 mA, with the stimulator turned on and the rheostat, which controls the amplitude, positioned at 0. The amplitude of stimulation was progressively increased at a rate of 0.6 mA/s until the patients reported the sensation of pricking. Particular attention was paid to avoid reaching the threshold of motor stimulation; if any movement of the investigated muscles was observed, the patient was excluded from the study. One patient with TMD and 1 control subject were excluded because the amplitude reached the motor threshold of the seventh cranial nerve, as shown by the contraction of the masseter and temporal muscles.

The same operator (RC) applied the pupillometer and delivered the TENS according to the manufacturer’s guidelines.

### Statistical Analysis

Statistical analysis was performed using STATA 10 (StataCorp LP, College Station, TX, USA) on average pupil sizes, computed on 60 seconds of recordings, as previously described [44]. Preliminary analysis of pupil size showed, as expected, a high correlation between the left and right pupil sizes, allowing the use of the left-right mean value to simplify the statistics. The ratio between pupil size in the presence of yellow-green light and that in darkness (herein referred to as the L/D ratio) was calculated. The Shapiro-Wilk test revealed a normal distribution of data. Within-group differences in the pupil size and L/D ratio were analyzed using a paired t test, while differences in pupil size between groups were analyzed using an unpaired t test. The level of significance was set at p = 0.05 for all tests. The results are expressed as the mean and standard deviation (SD).

## Results

The size of the pupil in darkness (infrared light) was significantly reduced during sensory low-frequency TENS both in the control (overall mean of 7886.51 before TENS vs. 7434.33 during TENS; p = 0.02) and TMD (7599.88 vs. 7148.94; p = 0.001) groups (Table 1). After TENS, the reduction of pupil size remained significant in the control group (7886.51 vs. 7427.11; p = 0.003) but not in the TMD group (7599.88 vs. 7490.61; p = 0.082) because of the return of pupil size to that prior to TENS.

**Table 1:** Comparison between the Pupil Size in the Control and TMD Groups in Infrared Light.

	Infrared pupil size	Infrared pupil size
	Control group	TMD group
Before TENS	7886.51 (1231.0)	7599.88 (1254.6)
During TENS	7434.33 (1464.0)*	7148.94 (1983.3)*
After TENS	7427.11 (1168.6)**	7490.61 (1477.6)

The data are expressed as the mean and standard deviation (in parentheses). Asterisks indicate statistically significant differences.

*paired t test within groups between the pre-TENS and TENS conditions: p = 0.02 in the control group and p = 0.001 in the TMD group.

**paired t test within groups between the pre-TENS and post-TENS conditions in the control group: p = 0.003.

The unpaired t test did not reveal any significant difference in pupil size between the control and TMD groups before TENS (7886.51 vs. 7599.88; p = 0.24), during TENS (7434.33 vs. 7184.94; p = 0.11), or after TENS (7427.11 vs. 7490.61; p = 0.47). In the TENS condition, it is possible that the higher value of data dispersion in the TMD group (SD 1983.31) was responsible for the lack of significance.

Table 2 shows the statistics of pupil size under yellow-green light. The size of the pupils in the control group was significantly reduced during TENS compared with the size prior to TENS (3934.98 vs. 3523.55; p = 0.005), and the pupil size continued to decrease post-TENS (3934.98 vs. 3294.16; p = 0.005). The TMD group did not show significant differences in pupil size between the pre-TENS and TENS conditions (2911.51 vs. 2836.38: p = 0.21) or between the pre-TENS and post-TENS conditions (2911.51 vs. 2973.83: p = 0.34). The unpaired t test between groups showed that the pupil size in the TMD group was significantly lower than that in the controls in the pre-TENS (2911.51 vs. 3934.38: p = 0.001) and TENS (2836.38 vs. 3523.55: p = 0.02) conditions, but no significant differences between groups were found in the post-TENS condition (2973.83 vs. 3294.16: p = 0.14). It is possible that, as in the post-TENS condition in darkness, the higher dispersion of data in the TMD group was responsible for the lack of significance. Note that the dispersion of data in the control group was lower than that in the TMD group, and, in the post-TENS condition, it was approximately one-third of that in the TMD group (sd: 617.32 vs. 1978.37). The absolute value of size differed by 10% (control 3294.16 vs. TMD 2973.83), suggesting more homogeneous pupil behavior among the control subjects.

**Table 2:** Comparison between Pupil Size in the Control and TMD Groups in the Yellow-Green Light Condition.

	Yellow-Green pupil size	Yellow-Green pupil size
	Control group	TMD group
Before TENS	3934.38 (821.35)	2911.51 (1041.97)°
TENS	3523.55 (867.38)*	2836.38 (1184.54)°°
After TENS	3294.16 (617.32)**	2973.83 (1978.37)

The data are expressed as means and standard deviations (in parentheses). Asterisks and circles show the statistically significant differences.

*paired t test within groups between the pre-TENS and TENS conditions in the control group: p = 0.003.

**paired t test within groups between the pre-TENS and post-TENS conditions in the control group: p = 0.0005.

°unpaired t test between the groups before TENS: p = 0.001.

°°unpaired t test between the groups during TENS: p = 0.02.

Results of the L/D ratio within and between groups are shown in Table 3 in the pre-TENS, TENS, and post-TENS conditions. The ratio decreased significantly in the control group pre-TENS compared with TENS (0.504 vs. 0.478: p = 0.03) and pre-TENS compared with post-TENS (0.504 vs. 0.444: p = 0.01). The TMD group did not show statistically significant differences in this ratio during the within-group comparisons.

**Table 3:** Comparison of the Pupil Size and Yellow-Green/Infrared Light (L/D) Ratio between the Control and TMD Groups.

	L/D ratio	L/D ratio
	Control group	TMD group
Before TENS	0.504 (0.111)	0.370 (0.091)°
TENS	0.478 (0.096)*	0.387 (0.123)°°
After TENS	0.444 (0.063)**	0.402 (0.112)

The data are expressed as means and standard deviations (in parentheses). Asterisks and circles show the statistically significant differences.

*paired t test within groups between the pre-TENS and TENS conditions in the control group: p = 0.03.

**paired t test within groups between the pre-TENS and post-TENS conditions in the control group: p = 0.01.

°unpaired t test between the groups before TENS: p = 0.0002.

°°unpaired t test between the groups during TENS: p = 0.01.

The L/D ratio was significantly higher in the control group compared with the TMD group, both pre-TENS (0.504 vs. 0.370: p = 0.0002) and during TENS (0.478 vs. 0.387: p = 0.01). Post-TENS, the L/D ratio remained higher in the control group (0.444 vs. 0.402), but the difference between the two groups was not significant (p = 0.08). Note that the ratio in the TMD group trended toward the value of the control group, with a value less than that in the TENS and pre-TENS conditions.

## Discussion

The data in our study can be summarized as follows:

1. No significant difference was found in pupil size in darkness between the control and TMD groups.
2. Significant within-group differences in pupil size in the darkness condition before TENS compared with during TENS in both the control and TMD groups were found; specifically, the pupil size was reduced during TENS.
3. Significant differences in pupil size in the darkness condition were found pre- compared with post-TENS in the control group but not in the TMD group. In the control group, the pupil size was reduced post-TENS, with the same reduction obtained during TENS; in the TMD group, the size of the pupil post-TENS returned close to the pre-TENS value.
4. Significant differences in pupil size in the light condition were found between the control and TMD groups; specifically, the TMD group showed a significantly smaller pupil size.
5. Significant within-group differences in pupil size in the light condition were found pre-TENS compared with during TENS and pre-TENS compared with post-TENS in the control group but not in the TMD group; pupil size in the light condition was reduced only in the control group.

Pupil size is a peripheral effect of a central network that controls the balance between sympathetic and parasympathetic outflow. The ambient light and the physiological and pathological causes of sympathetic excitation and/or parasympathetic inhibition evoke pupil dilation, and, vice versa, causes inducing sympathetic inhibition and/or parasympathetic excitation evoke pupil constriction. For this reason, pupil dynamics have been studied in physiological conditions that are suspected to impair autonomic balance and enhance arousal and sympathetic drive [61,62], enabling the assessment of the relationship between pupil dilation and noradrenergic activity [63,64]. Some authors have focused on the locus coeruleus-noradrenergic neuromodulatory system [65,66], which is considered to be one of the main centers regulating arousal, vigilance, alertness [67–70], pain, and sensory afferents, and it is likely involved in a variety of disorders, such as chronic pain and TMD, characterized by dysfunction of the noradrenergic arousal system [71–73].

**Fig 2:**
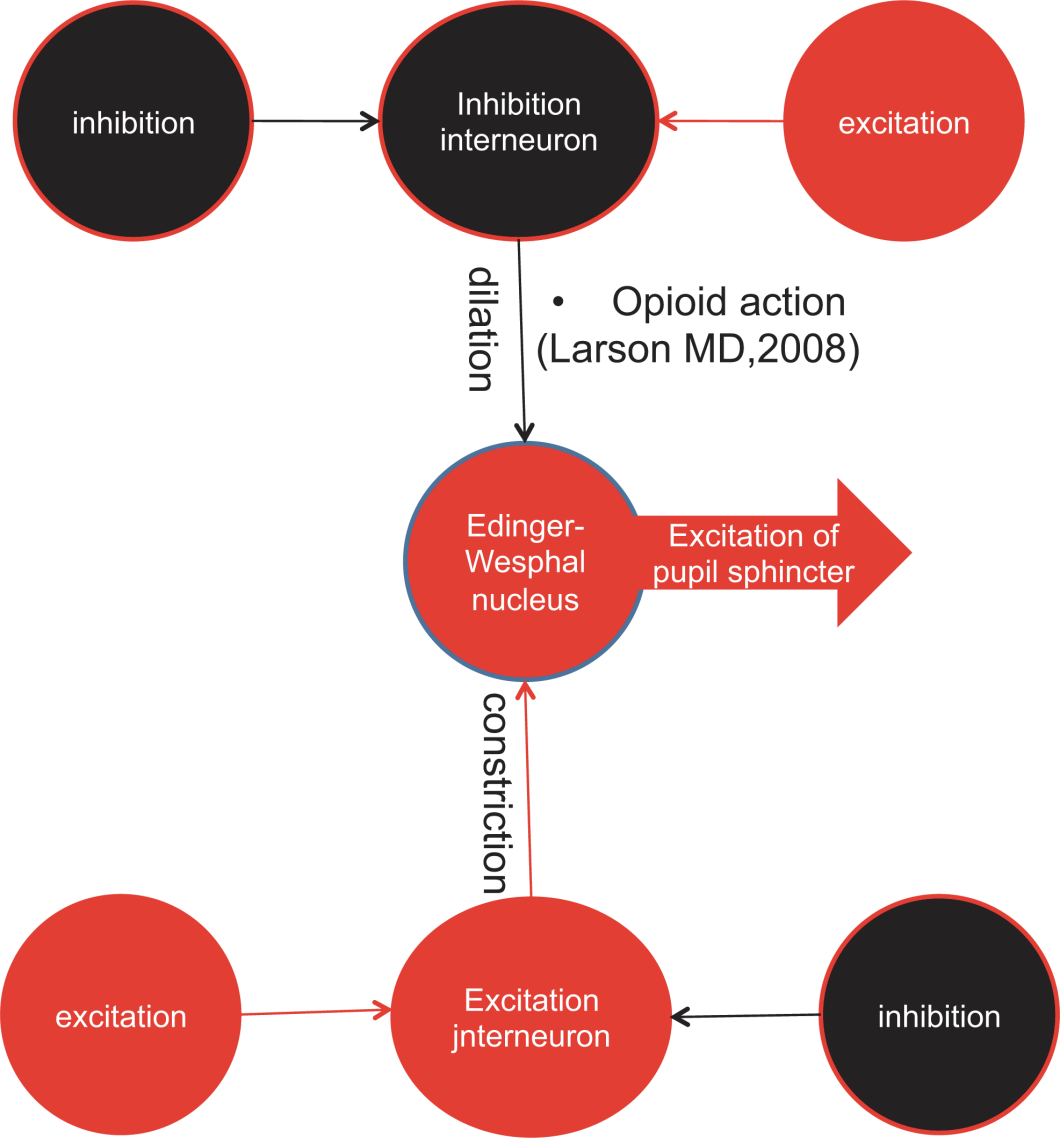
The Edinger-Westphal Nucleus (EWN) Exerts a Tonic Activity on the Pupil Sphincter. Larson [55] proposed that in anesthetized patients, opioid action can be exerted, blocking the efferents of inhibitory neurons to the EWN. Translating this hypothesis to non-anesthetized patients, it could be suggested that opioids reduce the inhibition of interneurons, increasing the effects of excitatory afferents of the EWN. The final effect was opioid miosis. In the figure, excitatory components of the model are shown in red, while inhibitory components are shown in black.

The locus coeruleus and the noradrenergic arousal/vigilance system receive opposing regulation by corticotropin-releasing factor and opioids [74,75]. Opioids reduce the activity of the locus coeruleus [76,77] and the diameter of the pupil [41–43,55]. In contrast, a wakefulness-promoting drug, likely acts on the locus coeruleus-noradrenergic system and induces pupil dilation [78,79] (Fig 2).

Low-frequency TENS induces a release of endorphins from the ventral-lateral PAG and the activation of the opioid descending path to the RVM. Simultaneously, PAG fibers extend to the pericoeruleus region, in which opioids are released in the dendritic system from the locus coeruleus neurons [80]. For this reason, it is possible that low-frequency TENS induces a reduction in the firing frequency in the locus coeruleus, simultaneously reducing the state of arousal, the perception of pain, and the diameter (size) of the pupil.

According to the above data, in our study, the pupil size decreased with low-frequency sensory TENS in darkness, both in the control and TMD groups. Five minutes after the end of stimulation, the effect was still present in the control group, but not in the TMD group.

The dilation of the pupil in darkness is mediated by the adrenergic sympathetic branch of the ANS and is supplied by a nerve originating from Budge’s cilio-spinal center that excites the dilator muscles of the pupil. The action of the dilator muscles is counterbalanced by the cholinergic parasympathetic branch, which originates from the Edinger-Westphal nucleus and inhibits the dilation. Moreover, pupil dilation obtained by sphincter inhibition can be equal to 1/3 of the maximum physiological dilation [81], and during mental or physical efforts, the pupil dilation may occur via central inhibition of the parasympathetic center [82].

It is possible that in the darkness condition in TMD patients, the endorphin effect due to TENS is sufficient to stimulate the parasympathetic inhibition of the dilator muscle; however, after the stimulation, if the baseline condition is characterized by impaired parasympathetic control, the stimulation provided by TENS rapidly completes its action on the pupillary muscles.

In the light condition, the parasympathetic branch responds to light that activates the sphincter muscle of the iris, causing miosis. At the same time, the sphincter receives beta-adrenergic innervation that is sufficient to reduce and/or counterbalance the contraction [83,84].

In our study, the subjects in the control and TMD groups responded to light as evidenced by a reduction in pupil size, demonstrating the proper activation of the central parasympathetic pathway that controls this basic function. Compared with the control group, the TMD patients showed a significantly higher reduction of pupil size, as indicated by both the absolute values and the ratio between the pupil size in light and in darkness. This finding might reflect impaired counterbalance of adrenergic inhibition or hyperexcitation of parasympathetic effectors in the TMD group. The result that TENS in the light condition reduces the size of the pupil in the control group but has no effect in the TMD group could suggest the impairment of the adrenergic counterbalance system, given that the opioid path works as an inhibitor of the parasympathetic branch of pupil control [55].

Taken together, the data from the present study show that TMD patients suffer from a type of impairment of the descending pathway that controls the sensory, pain, and autonomic output. It is possible that one of the mechanisms involved in this impairment can be addressed in the opioid descending pathway, considering that TMD patients have a weaker response to low-frequency sensory TENS than controls, with respect to modulating the inhibition of physiological parasympathetic excitation. This study focused on adrenergic dysfunction in TMD and confirmed previous studies in which TMD patients showed impairment in the recruitment of adrenergic activity in a weakly stressful task of muscle activation, such as forced voluntarily clenching [44] and other cognitive or emotional tasks [8].

**Fig 3:**
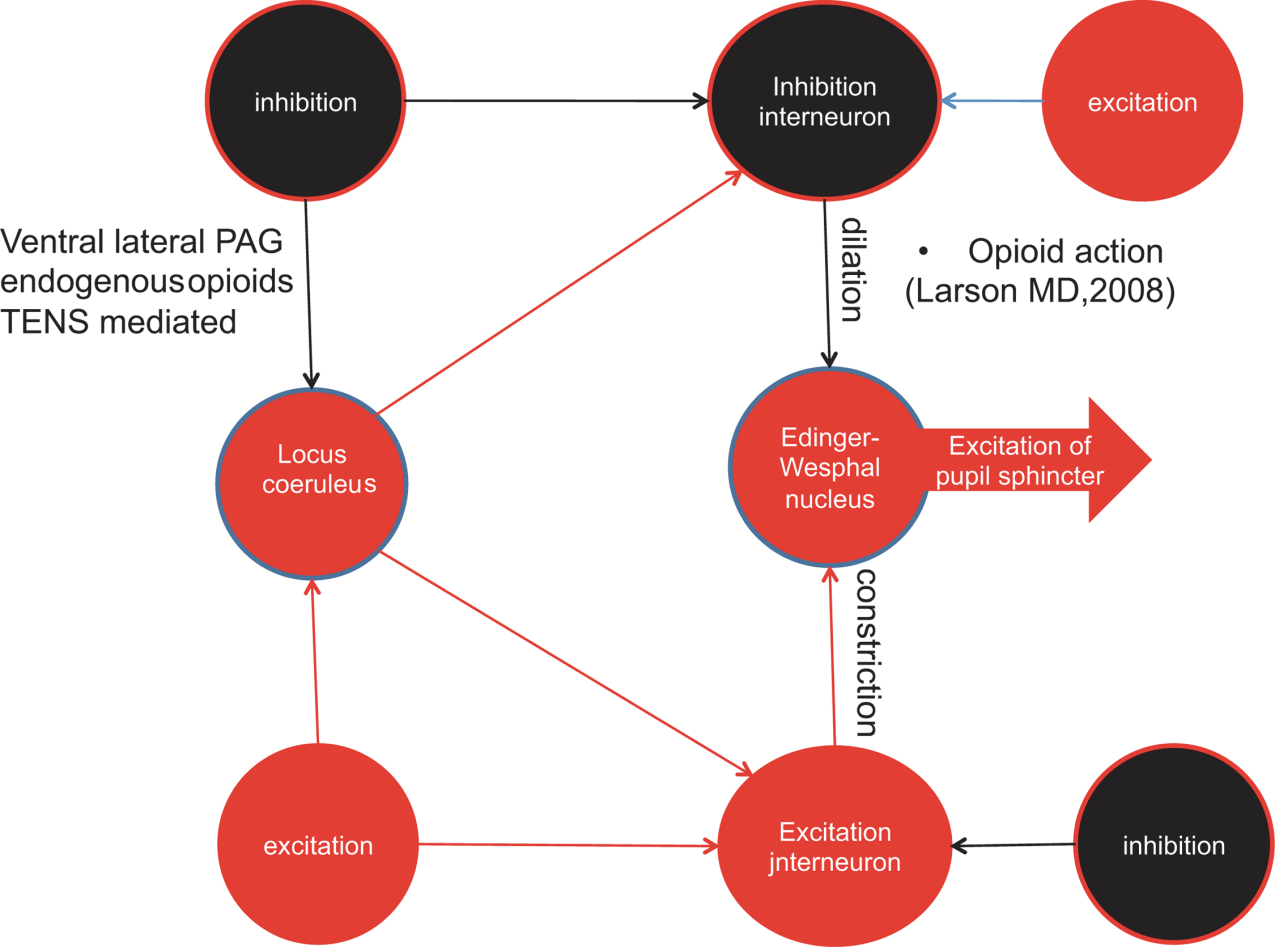
Mechanism of Endogenous Opioid Action Induced by LF TENS in Non-Anesthetized Subjects with Pupil Miosis. The locus coeruleus-noradrenergic system (LC) has been suggested to be a key structure in arousal, and its firing frequency tone has been correlated with wakefulness, vigilance, and sensory and pain perception. The size of the pupil correlates with the activity of LC, and light increases the frequency of firing. The action of the LC excites the excitation pathway that directly induces pupil constriction and excites inhibitory interneurons in the inhibitory pathway, exciting the inhibitor of the inhibitor; the net result is a reduction of inhibition and, indirectly, an agonist effect of the excitatory pathway of pupil constriction. Opioids act on the inhibitory pathway, and TENS has been suggested to be activated via the ventral lateral PAG and the opioid pathway. According to Valentino [74] and Curtis [75], opioids reduce the activation of the LC, and corticotropin-releasing hormone (CRH) increases the firing rate. It is possible that low-frequency sensory TENS inhibits LC inhibitory interneurons, increasing the tone of the pupillary constrictor of the LC. TMD patients could suffer from an impairment of the inhibitory pathway stemming from the ventral lateral PAG. Consequently, pupil constriction in response to light is not counterbalanced by inhibition (smaller pupil size under yellow-green light), and low-frequency sensory TENS is unable to further induce pupil constriction. Red, excitation pathway; black, inhibitory pathway.

It is probable that the impairments could be due to changes in the connectivity among the structures that belong to the distributed network of pain processing, such as the PAG, which is a node between cortical descending control and midbrain/spinal effectors [56,57,85,86] (Figs 2 and 3).

Confirmation of the involvement of the central descending pain pathway in future studies would explain the observation that therapies such as occlusal splint therapy, orthodontics, and physiotherapy yield short-term pain management success in only 75–80% of cases [87]. However, simple instruments are needed to test the impairment of the descending pathway. Konchak et al. [88] demonstrated that low-frequency TENS of the fifth and seventh pairs of cranial nerves “relaxed” only 75–80% of TMD subjects who received the stimulation. The remaining 20–25% showed signs of irritation after TENS, namely, an increase in EMG values of the jaw elevator muscles at rest and a lack of interocclusal freeway space. This result disagrees with the activation of the opioid descending system and directs attention to the activation of the sympathetic pathway, perhaps due to the stimulation of the dorsolateral PAG root.

As in a previous study [88], we used low-frequency TENS of the fifth and seventh pairs of cranial nerves to stimulate the descending control system and to examine the effect on the peripheral target. Beyond the absolute values of pupil size and the L/D ratio, it is noteworthy to mention that the data from TMD subjects are characterized by a higher standard deviation, an index of the dispersion of the values, compared with the controls. This higher dispersion of TMD data was particularly seen during and after TENS, while the dispersion was reduced in the control subjects. We speculate that low-frequency sensory TENS could aid in differentiating TMD patients with a higher impairment of the descending pain modulation pathway from those with a less impaired system. Unfortunately, we did not assess the response to TENS for the prediction of therapy. Thus, we were unable to determine whether different degrees of impairment, such as a lower light-darkness ratio or greater TENS reduction of the pupil size in the light condition, could predict the therapy outcome. Another limitation of our study is that we did not assess whether TENS induced a reduction in pain perception in the examined subjects; therefore, we could not directly assess the efficacy of our stimulation protocol on experimental pain. However, our goal did not concern the symptom “pain” and the efficacy of low-frequency TENS used in dentistry for its relief, and thus we did not take into account a specific study design to address the response to experimental pain. These topics are beyond the scope of the present work and are suggested for future studies.

Compared with the previous work [44], TMD patients in this study exhibited a smaller pupil size in darkness. The average patient age in the present study was 5.32 years older than that in the previous investigation. It is possible that this age discrepancy is responsible for this difference; in fact, it is known that pupil size decreases with age [49,50]. Moreover, it is possible that the duration of the disorder influences the degree of dysregulation, which shifts from a high sympathetic tone (greater pupil size in darkness) in the early stage of TMD to the failure of sympathetic counterbalance (lower pupil size in darkness) with the progression of the disorder. We did not assess the TMD duration in our sample; however, to assess the possible effect of this variable, the duration of the disorder must be investigated in the future through designed studies.

## Conclusion

The stimulation of the descending opioid pathway with low-frequency sensory TENS of the fifth and seventh pairs of cranial nerves and the evaluation of the effect on the peripheral target with a simple pupillometric parameter (pupil size) lead to the conclusion that TMD patients suffer from an impairment in the modulation of the descending pain system. Further studies are necessary to confirm this hypothesis and to relate the impairment of the descending pain system to the therapeutic outcome.
